# Bioactive Properties of Peptides Obtained from the Enzymatic Hydrolysis of Mesquite (*Prosopis laevigata*) Cotyledon Proteins

**DOI:** 10.3390/foods15081399

**Published:** 2026-04-17

**Authors:** Omar Sanchez-Jimenez, Erick Huerta-Rodriguez, Maria del Refugio Rocha-Pizaña, Diego A. Luna-Vital, Marco A. Mata-Gómez

**Affiliations:** Tecnologico de Monterrey, School of Engineering and Sciences, Ave. Eugenio Garza Sada 2501 Sur, Col: Tecnologico, Monterrey 64700, NL, Mexico; a01324800@tec.mx (O.S.-J.); erick.h.rodriguez@tec.mx (E.H.-R.); mrochap@tec.mx (M.d.R.R.-P.); dieluna@tec.mx (D.A.L.-V.)

**Keywords:** bioactive peptides, *Prosopis laevigata*, antioxidant, antimicrobial, anticancer, enzymatic inhibition

## Abstract

The identification of novel natural sources of bioactive peptides with multifunctional health-promoting properties remains a major challenge for the development of nutraceutical and therapeutic agents. *Prosopis laevigata* (mesquite), a plant of economic, medicinal, and nutritional relevance in Mexico, has been poorly explored as a source of protein-derived bioactive molecules. Therefore, this study evaluated the antioxidant, antimicrobial, cytotoxic, and enzymatic inhibitory activities of peptides obtained from the enzymatic hydrolysis of *P. laevigata* cotyledon proteins. The resulting hydrolysates exhibited significant antioxidant activity, for peptide fractions smaller and larger than 5 kDa, in the ABTS and FRAP assays. Cytotoxic activity against HepG2 liver cancer cells was observed at high peptide concentrations (8 mg/mL). Additionally, the peptides inhibited the growth of *Staphylococcus aureus* but showed no activity against *Escherichia coli*. The peptides also displayed partial inhibition of α-amylase activity, with peptides <5 kDa exhibiting competitive inhibition and peptides >5 kDa showing a mixed inhibition pattern. Overall, these findings highlight *P. laevigata* seeds as a promising source of multifunctional bioactive peptides with potential applications in functional foods and health-related biotechnological developments.

## 1. Introduction

Bioactive peptides (BPs) have gained considerable attention in food science due to their ability to exert beneficial physiological effects beyond basic nutrition. These peptides, typically released from food proteins through enzymatic hydrolysis or fermentation, have been associated with antioxidant, antimicrobial, anticancer, and enzyme-inhibitory activities, among others [1]. As a result, BPs are increasingly investigated as functional ingredients for the development of functional foods and nutraceutical products. However, the identification of novel and sustainable protein sources, as well as the comprehensive evaluation of their derived bioactivities, remain key challenges in this field. Plant-derived proteins, particularly those obtained from seeds, represent attractive substrates for BP production due to their high protein content, wide availability, and suitability for large-scale processing. In this context, seeds from leguminous plants have been extensively explored as precursors of bioactive peptides, since enzymatic hydrolysis can enhance protein digestibility and release peptides with relevant functional properties [2].

The genus *Prosopis* comprises approximately 44 species, of which 40 are native to the Americas, spanning from North to South America, while the remaining species originate from South Asia and Africa [3,4]. Commonly referred to as mesquite, these species are adapted to extremely arid and semi-arid environments characterized by low rainfall and atmospheric humidity, where they play an important ecological role through biomass production, soil enrichment, and atmospheric nitrogen fixation. In addition to their environmental relevance, *Prosopis* species represent valuable resources for human populations and livestock in resource-limited regions [5].

Mesquite trees provide durable wood for furniture, cooking, and heating, while their pods are used as cattle feed to lower livestock costs [6]. Traditionally, mesquite has been used to treat ailments such as malaria, asthma, fever, diarrhea, and liver infections [7]. Moreover, the pods are good sources of macronutrients. For instance, crude protein in *Prosopis alba* cotyledons represents approximately 62% of cotyledon flour, whereas the pod itself is a rich source of carbohydrates (75%) and fiber (35%) [5,8]. The highest concentration of proteins is found in the seeds, constituting about 60% of the total weight of the seed. The abundance of the main storage proteins present in the seed varies between species, with albumin and globulin being the most abundant with values from 18% to 44% and 30% to 60%, respectively, and to a lesser extent prolamin and glutelin [9,10].

Despite the nutritional relevance of *Prosopis* proteins, research focused on their bioactive properties, particularly at the peptide level, remains limited. While antioxidant peptides have been reported from enzymatically hydrolyzed seed proteins of *Prosopis alba* [8], and antimicrobial proteins have been described in Prosopis cineraria [11], there are currently no studies addressing the bioactive peptides derived from *Prosopis laevigata*. This species is widely distributed throughout Mexico and holds significant socioeconomic importance; however, its potential as a source of functional bioactive peptides has not been explored.

From a food science perspective, evaluating the bioactivities of peptides derived from *P. laevigata* proteins is highly relevant. Antioxidant peptides may contribute to oxidative stress mitigation, while antimicrobial peptides can support food safety and preservation [12,13]. Additionally, peptides capable of inhibiting digestive enzymes such as α-amylase are of interest for the development of functional foods aimed at modulating carbohydrate metabolism. The assessment of cytotoxic effects against cancer cell lines also provides preliminary insight into the biological activity and safety profile of these peptides.

Given this knowledge gap, the aim of the present study was to evaluate the antioxidant, antimicrobial, cytotoxic, and enzyme-inhibitory activities of bioactive peptides obtained from the enzymatic hydrolysis of *P. laevigata* cotyledon proteins. We hypothesized that enzymatic hydrolysis of *P. laevigata* seed proteins would generate peptide fractions with multifunctional bioactive properties, similar to those reported in other leguminous species. By providing the first report on the multifunctional bioactivity of peptides derived from this endemic species, this work contributes to the expanding body of research on plant-based bioactive peptides and highlights *P. laevigata* as a promising candidate for future applications in functional foods and food-related biotechnological developments.

## 2. Materials and Methods

### 2.1. Plant Material

Ripe *P. laevigata* pods were sourced from Xuchil Natural Products, a Mexican company specializing in mesquite and other natural products (State of Oaxaca, Mexico, Latitude: 17°48′ N, Longitude: 96°57′ W), on 17 April 2023. Following this, the proximate analysis of the pods and seeds samples of *P. laevigata* was performed [14].

### 2.2. Seed Flour Preparation

*P. laevigata* seed flour was prepared as follows [8]. The pods were dried at 50 °C until constant weight was reached. Dried pods were ground and sieved with a 2 mm mesh to separate seeds from the exocarp/mesocarp. Seeds were further ground to release the cotyledon and sieved with a 0.18 mm mesh. The flour was defatted using hexane (1:4, m/v) and stirred with a Cimarec+ stirring hotplate (Thermo Fisher Scientific, Waltham, MA, USA) for 24 h at 550 rpm. The hexane was removed by decantation. Then, the flour was washed twice with hexane and the suspension containing flour was centrifuged at 3000 *g* for 10 min (5702-RTM, Eppendorf, Framingham, MA, USA), dried in a fume hood for 36 h and stored in hermetic plastic bags at room temperature.

### 2.3. Protein Extraction

The overall experimental workflow involved the alkaline extraction of cotyledon proteins, simulated gastrointestinal enzymatic hydrolysis, and molecular weight-based peptide fractionation by ultrafiltration prior to bioactivity evaluation. Prior to alkaline extraction, the isoelectric point (pI) of *P. laevigata* cotyledon proteins was determined to maximize protein yields.

#### 2.3.1. Determination of the pI of *P. laevigata* Cotyledon Proteins

For this procedure, the methodology of Cattaneo et al. [8] was followed, with some modifications. Cotyledon flour was dissolved in water (1:10, m/v), adjusted to pH 9 with 0.5% (w/v) sodium hydroxide NaOH, stirred for 1 h, and centrifuged at 4400 *g* for 30 min at 4 °C. The pellet was discarded, and the supernatant was centrifuged again at 17,200 *g* for 20 min at 4 °C (Prism C2500R, Labnet International, Edison, NJ, USA ). Protein samples from the supernatant (1 mg/mL) were adjusted to pH values 2–12 with glacial acetic acid. After overnight incubation at 4 °C and further centrifugation, the protein concentration was determined according to the Bradford method [15], and the solubility percentage was calculated by using Equation (1).

(1) $$Solubility(\%)\;=\;\frac{Cf}{Ci}\times 100$$

where $C_{f}$ represents the soluble protein concentration of the supernatant after adjusting the pH, and $C_{i}$ is the soluble protein concentration of the sample before the acid/alkali treatment (1 mg/mL).

#### 2.3.2. Alkaline Extraction Assisted with Ultrasound

The protein extraction from the *P. laevigata* cotyledon flour was evaluated using both simple alkaline extraction and ultrasound-assisted alkaline extraction. Cotyledon proteins were extracted as previously described [8] with slight modifications. The cotyledon flour was dissolved in distilled water in a 1:10 (m/v) ratio maintaining constant stirring for 1 h at pH 10. The suspension was then subjected to ultrasound treatment using a probe sonicator (Model 250, Branson Ultrasonics, Danbury, CT, USA), operating at 70% amplitude for 15 min in pulse mode (5 s on/5 s off) while maintaining the sample in an ice bath to prevent overheating. Following this, the sample was centrifuged first at 4400 *g*, then the supernatant at 17,200 *g* for 30 min at 4 °C. The supernatant protein content was compared to a non-ultrasound control to evaluate the effect of cavitation on protein yield.

### 2.4. Protein Concentration

The protein extract was microfiltered and concentrated by using a 0.2 µm membrane set in a VIVAFLOW 200 filtration unit (Sartorius, Göttingen, Germany) until 50 mL were recovered in the retentate. The concentrated sample was diluted at a 1:5 ratio with distilled water and brought to pH 5 (pI). After overnight precipitation at 4 °C, the protein was centrifuged (3000 *g*, 60 min), then the pellet was frozen for 48 h and lyophilized (FreeZone 4.5 L Benchtop Freeze Dry System, Labconco, Kansas City, MO, USA) for storage at −20 °C until further use.

### 2.5. Electrophoresis Analysis

The protein profile was analyzed by SDS-PAGE, while peptides derived from the hydrolysis were analyzed by Tricine-SDS-PAGE. Briefly, protein molecular weight distribution from extract without hydrolysis was determined by SDS-PAGE [16]. In doing so, samples ranging from 1 to 8 µg/µL were loaded into a 15% polyacrylamide gel. Protein samples were resolved at 200 V for 45 min performed using a Mini-PROTEAN Tetra system (Bio-Rad Laboratories, Hercules, CA, USA). The polypeptide profile of the protein hydrolysate was analyzed as follows [17]. Electrophoresis was performed on a 16% T, 6% C monomer acrylamide separating gel with a 4% acrylamide stacking gel. For the sample preparation, cotyledon hydrolysate was diluted from 1:1 to 1:5 with distilled water, and then 10 μL of each sample was combined with 10 μL Laemmli buffer. The protein samples were resolved at 200 V for 60 min. The proteins were visualized by Coomassie staining.

### 2.6. FTIR Spectroscopy

FTIR was used to assess the quality and secondary structures of the protein isolate. An absorbance scan was performed from 4500 to 650 cm^−1^ at a resolution of 4 cm^−1^, employing a Shimadzu IRTracer-100 with QATR 10 attachment (Shimadzu Corporation, Kyoto, Japan). The spectra were deconvoluted using Origin Lab software (Version 10.1, OriginLab Corporation, Northampton, MA, USA) analyzing the amide I region (1600–1699 cm^−1^) by the second derivative approach. The smoothing of the data was performed by the Savitzky–Golay filter, with polynomial order 2, and 8 window points as input parameters. The Multiple Peak Fit function was used using the Gaussian method. The position of the secondary structures was based on previous reports [18,19,20], and the relative abundance was measured based on the area under the curve.

### 2.7. Enzymatic Hydrolysis

In vitro gastrointestinal digestion was performed using the method of Cattaneo et al. [8]. Briefly, lyophilized protein was dissolved in 50 mM citrate–phosphate buffer (2% m/v, pH 2.5). The mixture was incubated at 37 °C and agitated for 2 h. Thereafter, porcine pancreatin was added to the solution (enzyme: substrate, 1:20 w/w), the pH was adjusted to 7 and the mixture was incubated at 37 °C for another 2 h. The digestion was terminated by raising the temperature to 80 °C for 20 min.

### 2.8. Peptide Fractionation

The peptide mixture was separated using a VIVAFLOW 200 ultrafiltration unit (Sartorius, Göttingen, Germany) equipped with a 5 kDa molecular weight cut-off (MWCO) membrane. This process yielded two fractions: the retentate, containing peptides larger than 5 kDa, and the filtrate, containing peptides smaller than 5 kDa. Both fractions were frozen at −80 °C for 48 h, then lyophilized for another 48 h. The resulting dry peptides were stored at −20 °C until further use. This fractionation strategy allowed a direct comparison between low- and high-molecular-weight peptide populations to evaluate size-dependent bioactivity.

### 2.9. Antioxidant Assays

#### 2.9.1. ABTS Scavenging Activity

The ABTS scavenging activity of the fractionated peptides was measured as described by Liu et al. [21]. A stock solution (7 mM ABTS and 2.45 mM potassium persulfate) was prepared and incubated for 12 h in darkness. This solution was then diluted with 0.1 M phosphate buffer (pH 7.4) until absorbance was 0.75–0.8 at 734 nm. Subsequently, 10 µL of each peptide fraction solution at different concentrations (30, 60, 90, 120, 150 µg/mL) was mixed with 190 µL of the ABTS reagent and incubated for 10 min in the dark. Absorbance at 734 nm was measured in a Cytation 5 microplate spectrophotometer (BioTek Instruments, Winooski, VT, USA, and inhibition percentages were calculated as indicated in Equation (2).

(2) $$Inhibition(\%)=\frac{Control\;absorbance-Sample\;absorbance}{Control\;absorbance}\times 100$$

where *Control absorbance* corresponds to the absorbance of phosphate buffer instead of the sample, and *Sample absorbance* corresponds to the absorbance of each peptide concentration.

#### 2.9.2. Ferric-Reducing Antioxidant Power Assay

The ferric-reducing antioxidant power (FRAP) assay was conducted as previously described [22]. Peptide solutions were prepared at concentrations of 200, 400, 600, 800, and 1000 µg/mL in 0.2 M phosphate buffer (pH 6.6). For each sample, 960 µL was mixed with 1 mL of phosphate buffer and 1mL of 1% m/v potassium ferrocyanide. After mixing, the sample was incubated at 50 °C for 20 min and then cooled. Following this, 1 mL of 10% (m/v) TCA solution was added, mixed, and centrifuged (3220 *g*, 10 min). The supernatant was combined with 1 mL of distilled water and 0.2 mL of 0.1% ferric chloride (m/v). Absorbance was measured after a 10 min incubation at 700 nm in a microplate spectrophotometer (Cytation 5, Agilent Biotek, CA, USA). The reducing power of the peptides was calculated according to Equation (3).

(3) Reducing power=Sample absorbance−Control absorbance

where *Sample absorbance* corresponds to the absorbance of each peptide concentration, and *Control absorbance* corresponds to the absorbance of the phosphate buffer instead of the sample.

### 2.10. Cell Viability Assay

The antiproliferative potential of the peptides against HepG2 liver cancer cells was evaluated according to Flores-Cabrera et al. [23], with minor modifications. The cells were cultured in Dulbecco’s Modified Eagle Medium (DMEM) medium with 10% (v/v) fetal bovine serum (FBS) and 1% (v/v) penicillin–streptomycin (10,000 U/mL) at 37 °C in a 5% CO_2_ atmosphere. Cells (1 × 10^4^ cells/mL) were seeded in a 96-well plate and incubated for 24 h. Cells were then treated with peptide fractions at increasing concentrations (2, 4, 6, and 8 mg/mL) for 24 h; a cisplatin solution was used as a positive control at increasing concentrations between 37.5 and 600 µM. Cell viability was assessed using the 3-(4,5-dimethylthiazol-2-yl)-5-(3-carboxymethoxyphenyl)-2-(4-sulfophenyl)-2H-tetrazolium (MTS) assay (CellTiter 96 Aqueous One). Absorbance was read in a microplate spectrophotometer (Cytation 5, Agilent Biotek, CA, USA) at 490 nm to calculate cell viability (Equation (4)).

(4) $$Cell\;viability\left(\%\right)=\frac{Sample\;absorbance}{Control\;absorbance}\times 100$$

*Sample absorbance* corresponds to the absorbance of cells treated with the different concentrations of the peptides, and *Control absorbance* corresponds to the absorbance of cells without peptide treatment.

### 2.11. Antimicrobial Assay

Antimicrobial activity tests were carried out using *Escherichia coli* (ATCC 25922) and *Staphylococcus aureus* (ATCC 25923) as microbial models [24]. Bacterial suspensions were prepared by resuspending 4–5 colonies from LB agar into sterile saline solution (0.9% m/v NaCl) and adjusting the turbidity to 1.5 × 10^8^ UFC/mL, comparing the cell suspension with a 0.5 McFarland standard solution. MH agar plates were inoculated uniformly, allowed to dry, and sterile 6 mm disks were placed on each of them. Then, 10 μL of peptide fractions (5 mg/mL) and 5 μL of ampicillin (100 mg/mL) as a positive control were applied to the disks. The plates were incubated for 24 h at 37 °C, after which inhibition zones were measured with a digital caliper.

### 2.12. Enzyme Inhibition Assay

The inhibition assay of α-amylase was determined by the DNS colorimetric method previously reported by Zhou et al. [25] with slight modifications. A solution was prepared by mixing 100 µL of peptides ranging from 0 to 2.5 mg/mL with 100 µL of α-amylase (1.1 U/mL) in phosphate buffer (pH 6.9), briefly vortexing, and incubating for 10 min. Starch solutions ranging from 0.32 to 1.95% (m/v) were then added, followed by a 10 min incubation. Subsequently, a 200 µL volume of DNS reagent [26] was added to the mixtures and incubated at 98 °C for 5 min. After cooling the samples in an ice bath for 1 min and diluting them with 500 µL of distilled water, the absorbance was measured at 540 nm in a microplate spectrophotometer (Cytation 5, Agilent Biotek, CA, USA). For the controls, 100 µL phosphate buffer replaced the peptide solution and the procedure was repeated as described above. A glucose calibration curve was used to estimate the reducing sugar concentrations. The enzyme kinetic parameters *v_max_* and *K_M_* were determined by using Lineweaver–Burk plots, while the inhibition constant, *K_i_,* was determined by using Dixon plots. The inhibition constant Ki′ was determined by plotting [S]/V vs. [I] [27].

## 3. Results and Discussion

### 3.1. Proximate Analysis

Before obtaining the peptides, proximate analysis was performed to evaluate which part of the pod contained the major amount of protein. The proximate analysis revealed compositional differences (*p* < 0.05) between the pods and seeds of *P. laevigata*, particularly in moisture, crude fiber, and protein content (Supplementary Table S1). Although the protein content in *P. laevigata* pods was not negligible (16.0 ± 0.8%), soluble and non-soluble carbohydrates were the main nutritional components. This is in concordance with previous findings on *Prosopis juliflora* and *Prosopis palida*, which demonstrated a high carbohydrate content in pods of those species [28]. Similarly, studies on *Prosopis alba* and *Prosopis chilensis* pod flours demonstrated a higher content of soluble sugars and dietary fiber compared to cotyledon flour [29,30]. In contrast, *P. laevigata* seeds revealed a much higher protein content (40%) compared to pods (16%). Previous studies corroborated that the seeds of the *Prosopis* genus are a potential source of protein. For instance, *P. alba* seeds present a protein content of 32% [31], while *P. chilensis* has a protein content up to 31% [32], and *P. juliflora* has a protein content of 35% [33]. The protein content of *P. laevigata* seeds was found to be comparable to soybean (*Glycine max*) (35–40%) [34] and greater than common bean (*Phaseolus vulgaris*) (17–25%) [35], both of which are considered high-nutritional-value legumes.

### 3.2. Protein Extraction

The solubility of *P. laevigata* proteins decreased significantly above pH 3, reaching a minimum at pH 5 (below 10% solubility) and peaks between pH 9–10 with approximately 80% solubility (Supplementary Figure S1). Similar results have been found for species like *P. alba* and *P. juliflora,* where the pI of the protein was found to be in the range of 4.5–5 and 4–4.5, respectively [8,36]. Vegetable proteins typically have a pI between pH 3.5 and 6.5 [37]; this value is mainly influenced by the composition of amino acids present in the protein. Considering that the pI of the protein isolated from *P. laevigata* is at an acidic pH, it could be inferred that there is a higher proportion of Glu and Asp compared to amino acids such as His, Lys and Arg [38]. However, it should be noted that modifications of the side chains and the structural conformation of the protein can also affect the pI of the proteins [39].

Ultrasound-assisted alkaline extraction significantly increased (*p* < 0.05) protein yield to 306.13 ± 7.90 mg protein/g cotyledon flour (Supplementary Figure S2), nearly doubling the yield from simple alkaline extraction (153.93 ± 17.58 mg protein/g cotyledon flour), aligning with findings by [8] for *P. alba* (175.71 ± 48.65 mg protein/g cotyledon flour). This enhanced extraction efficiency could be attributed to acoustic cavitation, which transfers the energy generated by sound waves into the formation of bubbles within the intracellular medium. Upon collapse, the bubbles release the energy as temperature and pressure, resulting in cell disruption [40] and freeing the intracellular content in the process. Moreover, it has been proposed that ultrasonic wave treatment increases protein solubility by exposing hydrophilic amino acids to water, which increases interactions between the protein and the aqueous medium [41]. Therefore, proteins extracted by ultrasonic wave treatment were further used for hydrolysis.

### 3.3. Relative Molecular Weight Profile of Proteins and Peptides

**Figure 1:**
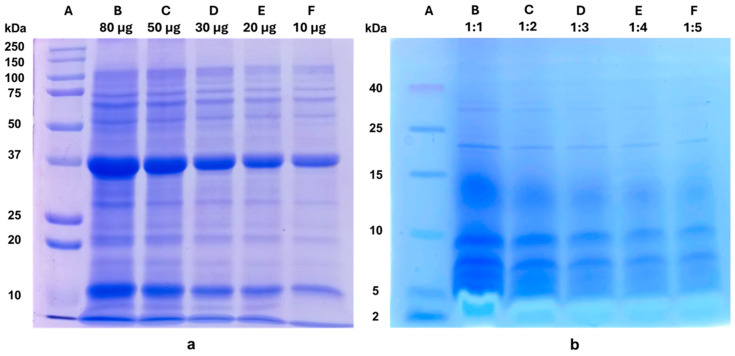
(**a**) SDS-PAGE gel of protein isolates from *P. laevigata* cotyledon flour; Lane A: molecular weight marker. Lanes B–F: protein isolate in loads corresponding to 80, 50, 30, 20 and 10 µg, respectively. (**b**) Tricine-SDS-PAGE gel of hydrolyzed protein isolates from *P. laevigata* cotyledon flour; Lane A: molecular weight marker. Lanes B–F: protein loads correspond to 1:1, 1:2, 1:3, 1:4 and 1:5 dilution of the hydrolyzed cotyledon flour proteins.

The protein profile of *P. laevigata* revealed a range of bands spanning from 15 kDa to 100 kDa (Figure 1a), with two prominent proteins around 37.42 kDa and 11.12 kDa. Additional bands were detected at 20.81, 28.46, 56.40, 66.91, and 72.88 kDa according to the relative mobility of the proteins (Supplementary Figure S3). This pattern closely resembles that of *P. alba*, whose SDS-PAGE profile spans 14 to 90 kDa, with key bands at 16, 38, 55, 67, and 85 kDa [31]. These similarities suggest possible genomic conservation, although further genomic analysis is needed to confirm homology. The strong bands observed at approximately 37.42 kDa and 11.12 kDa may correspond to storage proteins such as vicilin-like proteins and 2S albumins, based on previously reported molecular weight ranges in leguminous species. For example, a 33 kDa vicilin-like allergenic protein has been reported in *P. juliflora* [42], and similar vicilin subunits are known in other legumes like peas and chickpeas [43]. The 11 kDa band likely corresponds to a 2S albumin, a prominent storage protein in ungerminated seeds that accounts for around 40% of total seed protein [44]. Notably, 2S albumins are precursors of around 18–21 kDa, which are processed into smaller subunits of 8 to 15 kDa [44,45,46]. After simulated digestion with pepsin and pancreatin, the molecular weight distribution shifted in the Tricine-SDS-PAGE profile (Figure 1b), with most bands now in the 10–25 kDa range, while a few remained between 25 and 40 kDa. This peptide profile aligns with findings by [8] for hydrolyzed cotyledon proteins from *P. alba*, where similar-sized peptides were observed. These results highlight the digestibility potential of *P. laevigata* proteins into smaller peptides, potentially increasing their bioavailability and bioactivity. Moreover, knowing the molecular weight profile of *P. laevigata* proteins supports further exploration of their functional properties, particularly their potential bioactive roles, to enhance their application in food science and health-related research.

### 3.4. Structural Analysis of Protein

**Figure 2:**
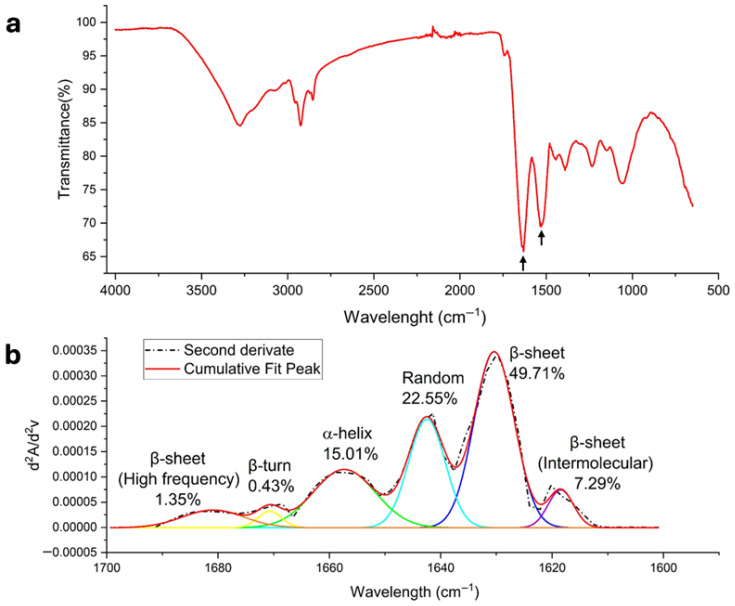
(**a**) Infrared spectrum of *P. laevigata* lyophilized proteins; from left to right, the position of amide I and amide II are indicated with black arrows. (**b**) Amide I band deconvolution in the 1700–1600 (cm^−1^) range depicting percentage of secondary structures.

The absorption spectrum (4500–450 cm^−1^) of *P. laevigata* lyophilizate showed a typical protein pattern (Figure 2a), with prominent amide II and I bands near 1700–1500 cm^−1^. Figure 2b shows that the β-sheet conformation was the most abundant secondary structure (49.71%), followed by random structures (22.55%) and α-helix (15.01%). The predominance of these structures depends on amino acid composition, as some residues preferentially form α-helices (e.g., Ala, Glu, Gln, Lys, Arg, Leu, Met), while others stabilize β-sheets (e.g., Val, Ile, Phe, Tyr, Thr, Trp, Cys) [47,48]. The dominance of β-sheets suggests a stable structure, which may impact protein digestibility and functional behavior, considering that secondary structure influences protein stability, solubility, and susceptibility to enzymatic hydrolysis [49]. Additionally, this analysis contributes to the limited structural characterization of mesquite proteins, reinforcing their potential for functional food or bioactive compound development.

### 3.5. Antioxidant Assays

**Figure 3:**
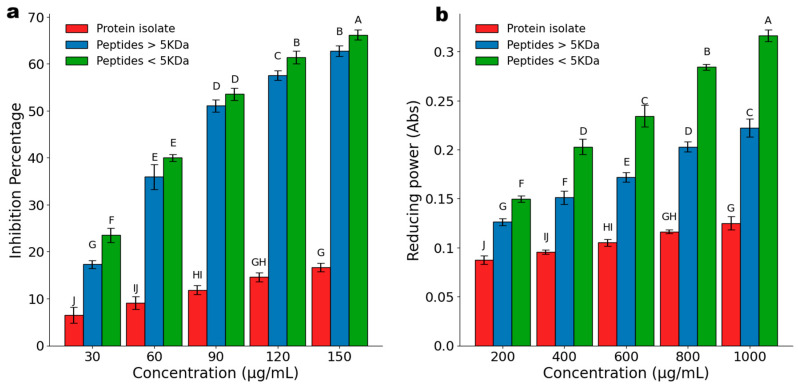
(**a**) ABTS scavenging activity of peptide fractions and protein isolate. (**b**) Changes in absorbance for both the peptide fractions and the protein isolate. A higher value of Abs means a more potent antioxidant effect. Means in a bar followed by the same letter are not significantly different (ANOVA, Tukey test, α = 0.05).

The antioxidant activity of *P. laevigata* protein hydrolysates was evaluated using ABTS radical scavenging and FRAP assays, which assess free radical neutralization and ferric-reducing capacity, respectively. In the ABTS assay (Figure 3a), both peptide fractions exhibited significantly higher antioxidant activity than the non-hydrolyzed protein isolate, confirming that enzymatic hydrolysis enhanced the release of antioxidant compounds. A clear dose–response relationship was observed for all samples. The <5 kDa peptide fraction showed the highest radical scavenging capacity, reaching 66% inhibition with an SC_50_ of 81.567 ± 0.59 µg/mL, whereas the >5 kDa fraction achieved 62% inhibition with an SC_50_ of 89.257 ± 2.75 µg/mL. In contrast, the protein isolate displayed limited activity, reaching only 16% inhibition at its highest tested concentration (150 µg/mL). Significant differences (*p* < 0.05) between peptide fractions were detected at 30, 120, and 250 µg/mL, while no significant differences were observed at intermediate concentrations (60 and 90 µg/mL).

The greater antioxidant capacity of the <5 kDa fraction was consistent with previous reports indicating that lower molecular weight peptides generally exhibit enhanced radical scavenging activity. Similar trends have been reported for *Prosopis alba*, where peptides smaller than 3 kDa showed markedly stronger antioxidant activity than higher molecular weight fractions [8]. When compared with other plant-derived protein hydrolysates, *P. laevigata* peptides demonstrated competitive antioxidant potential, surpassing those obtained from *Amaranthus mantegazzianus* (SC_50_ = 1.36 mg/mL), *Cucurbita moschata* seeds (SC_50_ = 142.3 µg/mL), and *Cicer arietinum* (SC_50_ = 1000 µg/mL) [50,51]. Similar size-dependent antioxidant effects have been reported in peptide fractions obtained from other biological sources. For instance, ref. [52] reported that lower molecular weight peptides extracted from silver carp scales exhibited enhanced radical scavenging capacity following membrane separation. Overall, these findings are consistent with previous observations in plant-derived protein hydrolysates, where low-molecular-weight peptides exhibit enhanced antioxidant activity due to their increased accessibility and interaction with reactive species.

FRAP assay results (Figure 3b) further corroborated the antioxidant potential of the hydrolysates. The <5 kDa peptide fraction reached an absorbance of 0.316 ± 0.008 at 1000 µg/mL, while the >5 kDa fraction achieved 0.222 ± 0.005 Abs. In contrast, the protein isolate showed limited reducing power (0.125 ± 0.006 Abs). Statistical analysis confirmed significant differences (*p* < 0.05) among peptide fractions and between hydrolysates and the protein isolate across the evaluated concentrations. Although direct comparisons with other *Prosopis* species are scarce, these findings align with studies on lentil (*Lens culinaris*) and common bean (*Phaseolus vulgaris*) hydrolysates, which also reported enhanced ferric-reducing capacity following enzymatic hydrolysis [53,54]. Additionally, the observed influence of peptide size on reducing power was consistent with reports on *Sphenostylis stenocarpa*, where smaller peptides exhibited superior electron-donating capacity compared to larger fragments [55].

The antioxidant activity observed in both assays may be attributed to the presence of specific amino acid residues known to promote radical scavenging and reducing reactions, including hydrophobic (Leu), basic (Lys, His), aromatic (Tyr), and sulfur-containing amino acids [56]. These residues facilitate interactions with reactive radicals and ferric ions, enhancing antioxidant performance [55]. In the FRAP assay, the reducing activity is mainly associated with redox-based electron donation mechanisms rather than metal chelation, For instance, amino acids such as Tyr, Trp, Met, Lys, and Cys can reduce Fe^+3^ to Fe^+2^, [12] further supporting the role of peptide composition and size in determining antioxidant behavior [55,57].

Although ABTS and FRAP assays do not fully replicate biological conditions, they are widely accepted as preliminary screening tools for assessing antioxidant potential in food-derived peptides. Therefore, the results obtained indicate that *P. laevigata* protein hydrolysates, particularly low-molecular-weight fractions, represent promising candidates for natural antioxidant applications. These findings are in agreement with the broader body of literature on plant-derived bioactive peptides, reinforcing the relevance of peptide size and composition in determining antioxidant activity. Further studies involving peptide sequencing, amino acid profiling, and in vitro or in vivo models are required to better elucidate structure–activity relationships and confirm their functional relevance in food systems.

### 3.6. Antiproliferative Effect of P. laevigata Peptides

**Figure 4:**
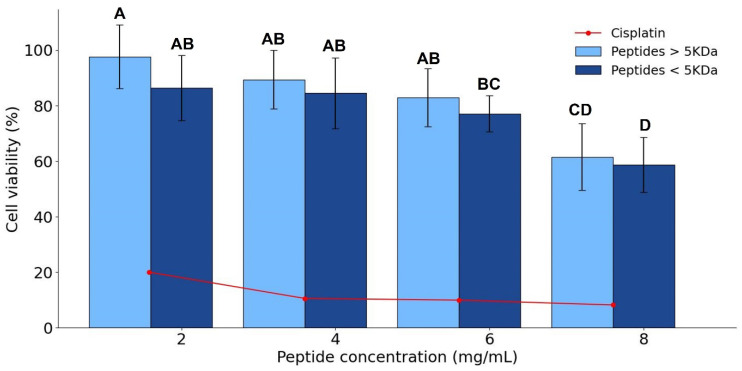
Cell viability assay against HepG2 cell line. Means in a bar followed by the same letter are not significantly different (ANOVA, Tukey test, α = 0.05).

The antiproliferative activity of *P. laevigata* peptides was evaluated using the MTS cell viability assay on HepG2 liver cancer cells. Both peptide fractions (<5 kDa and >5 kDa) induced a reduction in cell viability at the highest tested concentration (8 mg/mL), reaching 58.59 ± 9.85% and 61.49 ± 11.93% viability, respectively (Figure 4). The decrease in cell viability followed a dose-dependent trend, with statistically significant effects (*p* < 0.05) observed across concentrations according to post hoc analysis. However, no significant differences were detected between peptide fractions, indicating that molecular weight did not markedly influence antiproliferative activity under the evaluated conditions.

Although the antiproliferative effects were observed at relatively high concentrations, this behavior was consistent with previous reports indicating that HepG2 cells often display limited sensitivity to food-derived peptides at low doses. For example, corn gluten peptides (3–5 kDa) showed negligible cytotoxic effects on HepG2 cells at concentrations of 50 and 200 µg/mL, although delayed cell growth associated with S-phase arrest was reported [58]. Similarly, sorghum-derived peptides did not induce cytotoxicity in HepG2 cells within the 50–200 µg/mL range [59]. Comparable outcomes have been reported for peptides derived from goat milk casein and bovine α-lactalbumin, which failed to significantly reduce HepG2 viability at concentrations between 25 and 1000 µg/mL [60,61].

In contrast, collagen peptides derived from cowhide have demonstrated a more pronounced antiproliferative effect at concentrations comparable to those used in the present study, reducing HepG2 viability to approximately 80% at 5 mg/mL and to 22% at 11 mg/mL [62]. These differences highlight the strong influence of peptide origin, composition, and sequence on antiproliferative potency, and suggest that not all food-derived peptides exert comparable effects against cancer cell lines.

The moderate antiproliferative activity observed for *P. laevigata* peptides should be interpreted as preliminary bioactivity rather than as a direct anticancer effect. The results indicate that these peptides are not highly cytotoxic at low concentrations, which may be advantageous in terms of safety for food-related applications. Although the present assay provides preliminary insight into the antiproliferative potential of *P. laevigata* peptides, further studies would benefit from evaluating a broader concentration range, including non-cancerous cell lines to better assess the selectivity and biological relevance of the observed effects. Moreover, further studies focusing on peptide purification, sequence identification, and mechanisms of action involved (such as cell cycle analysis or apoptosis-related pathways) would be necessary to better understand the biological relevance of these effects and to optimize conditions for enhanced bioactivity.

### 3.7. Antimicrobial Assay

**Figure 5:**
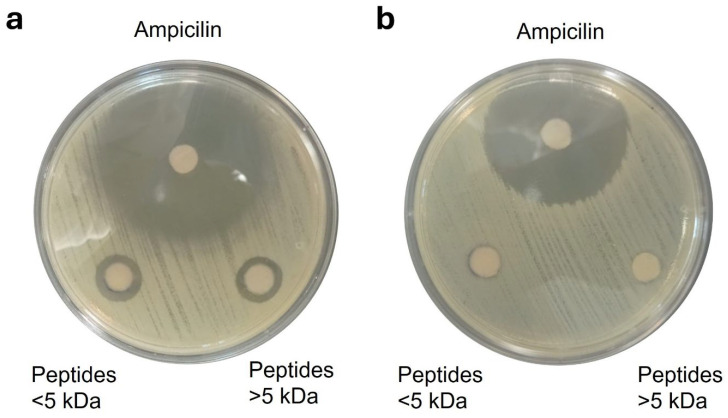
Antimicrobial test of mesquite peptides. (**a**) S. *aureus* and (**b**) *E. coli* strains were grown on MH agar inoculated with disks impregnated with 10 µL of each peptide fraction at 5 mg/mL.

The antimicrobial activity of *P. laevigata* peptides was evaluated using the Kirby–Bauer disk diffusion assay against *Staphylococcus aureus* and *Escherichia coli*. The results showed that peptide fractions inhibited the growth of *S. aureus* at a concentration of 5 mg/mL, with the <5 kDa fraction exhibiting the strongest inhibitory effect (Figure 5). Statistical analysis confirmed significant differences (*p* < 0.05) in inhibition zones between peptide fractions, indicating that peptide size played a relevant role in antimicrobial efficacy. In contrast, no inhibitory activity was observed against *E. coli* at the same concentration.

The susceptibility of *S. aureus* to *P. laevigata* peptides was consistent with previous studies reporting the effectiveness of plant-derived antimicrobial peptides against Gram-positive bacteria. For instance, previous reports demonstrated that antimicrobial peptides isolated from *Moringa oleifera* seeds effectively inhibited *S. aureus*, with minimum inhibitory concentration (MIC) values around 2 mg/mL, despite differences in peptide sequence and source [60,61]. These findings support the notion that plant-derived peptides can exert selective antimicrobial activity, particularly against Gram-positive pathogens.

The observed selectivity toward *S. aureus* can be explained by differences in bacterial cell envelope structure. Antimicrobial peptides can exert their killing effect by several mechanisms; however, the most common rely on their direct interaction with bacterial membranes [63]. Gram-positive bacteria possess a single lipid membrane surrounded by a thick peptidoglycan layer, which is more accessible to antimicrobial peptides. In contrast, Gram-negative bacteria such as *E. coli* contain an additional outer membrane rich in lipopolysaccharides, which acts as an effective permeability barrier and limits peptide penetration. Most antimicrobial peptides share common structural features, including α-helical conformation, net positive charge, and a substantial proportion of hydrophobic residues (40–50%), which facilitate electrostatic interactions with negatively charged bacterial membranes and subsequent membrane disruption [64,65].

The lack of antimicrobial activity against *E. coli* may also be related to peptide concentration and composition. Previous studies have reported that substantially higher concentrations (MIC > 80 mg/mL) are required to inhibit *E. coli* when treated with plant-derived hydrolysates or Maillard reaction products [66]. Additionally, peptide amino acid composition plays a crucial role in antimicrobial performance. Peptides enriched in acidic residues such as Glu and Asp tend to exhibit reduced antimicrobial potency, whereas those rich in basic residues such as Arg and Lys show enhanced activity due to stronger electrostatic interactions with bacterial membranes. Song et al. [67] reported that peptides with high acidic residue content may even promote *E. coli* growth, providing a plausible explanation for the resistance observed in the present study. Further studies involving a broader range of bacterial strains, peptide characterization, and determination of MIC values are necessary to fully elucidate their antimicrobial spectrum and mechanisms of action.

### 3.8. Enzyme Inhibition Assay

**Figure 6:**
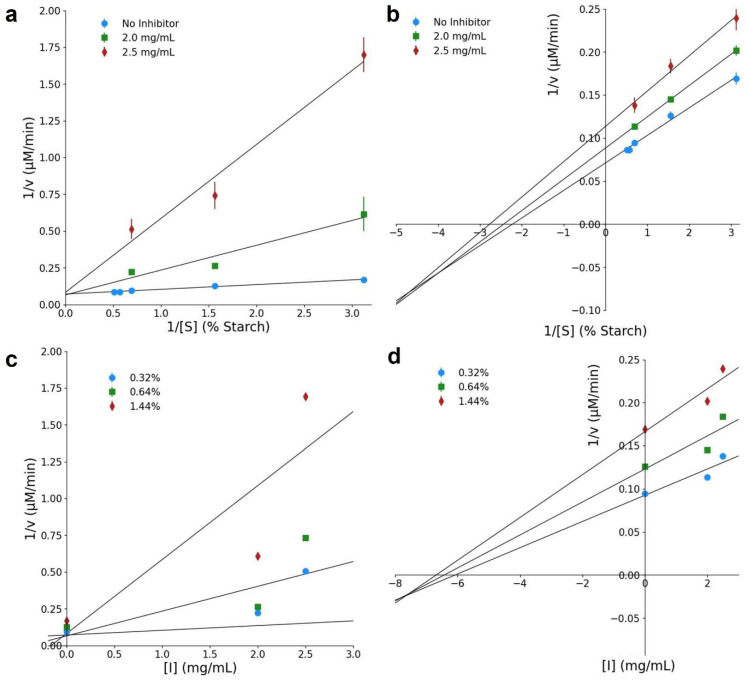
Double reciprocal Lineweaver–Burk plot for the inhibition of α-amylase in the presence of (**a**) peptides with MW > 5 kDa and (**c**) peptides with MW < 5 kDa. Dixon plot for (**b**) peptides with MW > than 5 kDa and (**d**) < than 5 kDa. Data are the mean of three independent experiments.

The inhibitory effect of *P. laevigata* peptides on α-amylase activity was evaluated through enzyme kinetics, revealing distinct inhibition mechanisms depending on peptide molecular weight. Peptides smaller than 5 kDa exhibited a competitive inhibition pattern, as evidenced by the Lineweaver–Burk plot (Figure 6a), where all regression lines intersected at the *y*-axis. In this case, the maximum reaction velocity $v_{max}$ remained constant, while the Michaelis–Menten constant ($K_{M}$) increased proportionally with inhibitor concentration (0.45–11.9 mg/mL), which is characteristic of competitive inhibition [68]. The inhibition constant $K_{i}$, determined using the Dixon plot (Figure 6b), further confirmed the affinity of low-molecular-weight peptides for the active site of α-amylase.

**Figure 7:**
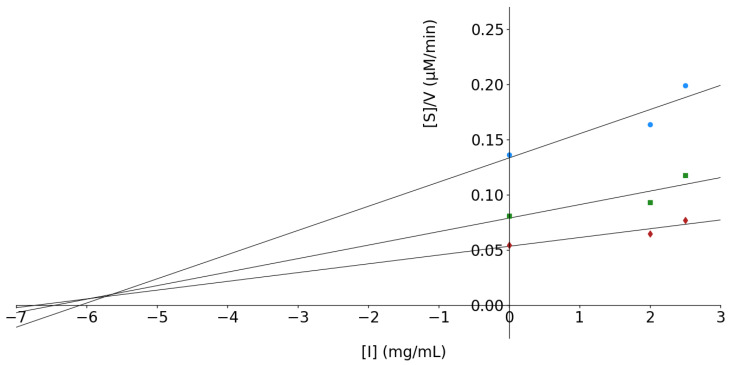
Cornish–Bowden plot for peptides with MW > than 5 kDa. The point where the lines intersect corresponds to an estimate of ${K_{i}}^{\prime }$ value. The concentration (m/v) of the substrate were ♦ (1.44%), ■ (0.64%), and ● (0.32%).

In contrast, peptides larger than 5 kDa followed a mixed-type inhibition mechanism. The Lineweaver–Burk plot (Figure 6c) showed a progressive decrease in $v_{max}$ values (from 14.00 to 8.80 µM/min) with increasing inhibitor concentration, while changes in $K_{M}$ suggested a tendency toward uncompetitive behavior [69]. This interpretation was supported by the inhibition constants obtained from the Dixon (Figure 6d) and Cornish–Bowden plots (Figure 7). The $K_{i}$ value (7.69 mg/mL) was higher than the ${K_{i}}^{\prime }$ value (5.81 mg/mL), indicating a stronger affinity of these peptides for the enzyme–substrate (ES) complex rather than for the free enzyme, thus confirming an uncompetitive contribution within the mixed inhibition pattern. A summary of all kinetic parameters derived from this analysis is presented in Table 1.

The distinct inhibition mechanisms observed between peptide fractions highlight the influence of peptide size on enzyme–inhibitor interactions. Low-molecular-weight peptides are more likely to access and interact directly with the catalytic site of α-amylase, resulting in competitive inhibition, whereas larger peptides may preferentially bind to secondary sites or stabilize the ES complex, leading to mixed or uncompetitive inhibition. This size-dependent behavior has also been reported for plant-derived peptides from other sources. For example, previous reports demonstrated that quinoa peptides smaller than 1 kDa exhibited strong α-amylase inhibition, with peptide MMFPH acting as a competitive inhibitor at low concentrations and shifting toward non-competitive inhibition at higher doses [22], while soybean peptides with lower molecular weights exhibited stronger inhibitory effects against α-amylase compared to heavier peptides [70]. Such occurrence in kinetic behaviors described for plant-derived peptides, where molecular weight and peptide–enzyme interactions influence competitive or mixed inhibition patterns, supports the relevance of kinetic analysis in functional food research [71].

Although the inhibitory potency of *P. laevigata* peptides was lower than that reported for some highly active peptide inhibitors, the observed $K_{M}$ values (3.76 mg/mL at 2 mg/mL inhibitor concentration) confirm their capacity to modulate α-amylase activity.

α-Amylase is a key enzyme in carbohydrate metabolism and represents an important target for controlling postprandial hyperglycemia. While synthetic inhibitors such as acarbose, miglitol, and voglibose are clinically used, they are often associated with gastrointestinal side effects. Consequently, bioactive peptides from natural sources have emerged as promising alternatives for the development of functional foods and nutraceuticals aimed at glycemic control [72,73]. In this context, the present study provides the first report of α-amylase inhibition kinetics for peptides derived from *Prosopis* species, positioning *P. laevigata* as a novel plant source of enzyme-modulating peptides with potential food-related applications.

## 4. Conclusions

Taken together, the results of this study demonstrate that enzymatic hydrolysis of *P. laevigata* cotyledon proteins generates peptides with a multifunctional bioactive profile, largely influenced by molecular weight. Across the evaluated assays, low-molecular-weight peptides (<5 kDa) generally exhibited enhanced bioactivity compared to larger fractions, highlighting peptide size as a critical factor governing functional performance.

The antioxidant activity observed through ABTS and FRAP assays indicates that *P. laevigata* peptides possess effective radical scavenging and electron-donating capacities, particularly within the low-molecular-weight fraction. These properties are relevant from a food science perspective, as oxidative processes are closely associated with food deterioration and chronic disease development. Similarly, the selective antimicrobial activity against *Staphylococcus aureus* suggests the potential application of these peptides as natural antimicrobials targeting Gram-positive bacteria, a behavior consistent with reported differences in bacterial cell envelope structure.

The α-amylase inhibition assays revealed that *P. laevigata* peptides can modulate enzyme activity through distinct inhibition mechanisms depending on peptide size. Competitive inhibition by smaller peptides and mixed or uncompetitive behavior by larger peptides indicate diverse enzyme–peptide interactions, supporting their potential role in moderating carbohydrate digestion. Such partial and mechanism-dependent inhibition is often desirable in functional food applications, as it may contribute to glycemic control without complete enzymatic suppression.

Regarding antiproliferative activity, *P. laevigata* peptides induced a moderate, dose-dependent reduction in HepG2 cell viability at high concentrations. Although limited at lower doses, this response aligns with reports for other food-derived peptides and suggests a favorable safety profile rather than a pharmacological anticancer effect.

Despite these promising findings, several limitations should be acknowledged. The study employed a single molecular weight cut-off (5 kDa) for peptide fractionation, which, while suitable for exploratory size-dependent evaluation, may overlook finer distinctions achievable with multiple cut-offs. In addition, peptide identification and sequencing by LC–MS/MS were not performed, limiting direct structure–activity correlations, which also highlights the way for further research. The antimicrobial assessment was also restricted to two representative bacterial strains, and broader screening could further elucidate the spectrum of activity, as well as a method that allows the determination of quantitative parameters such as minimum inhibitory concentration (MIC).

Overall, this work provides the first comprehensive evaluation of multifunctional bioactivities of peptides derived from *P. laevigata*. The findings support its potential as an underexplored source of bioactive peptides for functional food and nutraceutical applications, while establishing a foundation for future studies focused on peptide identification, structure–activity relationships, and validation in more complex biological or food systems.

## Figures and Tables

**Table 1:** Inhibition kinetics parameters for peptide fractions <5 and >5 kDa.

Peptide Size (kDa)	Kinetic Parameter	Inhibitor Concentration (mg/mL)		
		0	2	2.5
<5	*vmax* (µM/min)	14.00	14.61	13.65
	K_M_ (mg/mL)	0.45	3.77	11.9
	K_i_ (mg/mL)		0.25	
>5	*vmax* (µM/min)	14.00	11.31	8.80
	K_M_ (mg/mL)	0.45	0.41	0.36
	K_i_ (mg/mL)		7.69	
	K_i_′ (mg/mL)		5.81	

## Data Availability

The original contributions presented in the study are included in the article/Supplementary Materials; further inquiries can be directed to the corresponding author.

## Supplementary Materials

The following supporting information can be downloaded at https://www.mdpi.com/article/10.3390/foods15081399/s1: Table S1. Proximate analysis on a dry basis of the seeds and pods of *P. laevigata*. Figure S1. pH-solubility profiles of protein isolate from cotyledon flour. Figure S2. Comparison of alkaline extraction vs. ultrasound-assisted alkaline method. Figure S3. Plot of the relative mobility (Rm) of the protein with respect to the log of the molecular weight.

## Abbreviations

The following abbreviations are used in this manuscript:

ABTS	2,2′-Azino-bis(3-ethylbenzthiazoline-6-sulfonic Acid)
BPs	Bioactive peptides
DNS	3,5-dinitrosalicylic acid
FBS	Fetal bovine serum
FRAP	Ferric-reducing antioxidant power
FTIR	Fourier-transform infrared spectroscopy
MTS	3-(4,5-Dimethylthiazol-2-yl)-5-(3-carboxymethoxyphenyl)-2-(4-sulfophenyl)-2H-tetrazolium
SC_50_	Scavenging concentration 50
