# Supplementary material for: Bioactive Properties of Peptides Obtained from the Enzymatic Hydrolysis of Mesquite (Prosopis laevigata) Cotyledon Proteins
Source: Foods. 2026 Apr 17;15(8):1399. doi: 10.3390/foods15081399 (PMC13114964; doi:10.3390/foods15081399)
Supplement: Supplementary file 1 [file foods-15-01399-s001.zip › foods-4202341-supplementary.pdf]

Supplementary tables and images

Table S1. Proximate analysis on a dry basis of the seeds and pods of *P. laevigata*.

Sample	Moisture (%)	Crude protein (%)	Total fat (%)	Ash (%)	Crude fiber (%)	NFE (%)
Pod	10.97 ± 0.89[a]	16.02 ± 0.79[a]	4.06 ± 0.54[a]	3.55 ± 0.07[a]	15.32 ± 1.02[a]	50.24 ± 3.16[a]
Seed	4.93 ± 0.12[b]	40.30 ± 2.68[b]	3.71 ± 0.60[a]	3.18 ± 0.23[a]	4.97 ± 0.44[b]	43.05 ± 3.16[b]

NFE: Nitrogen Free Extract. The results are expressed as the average of the samples ± standard deviation. Means in a column followed by the same letter are not significantly different (two-sample t-test, α=0.05).

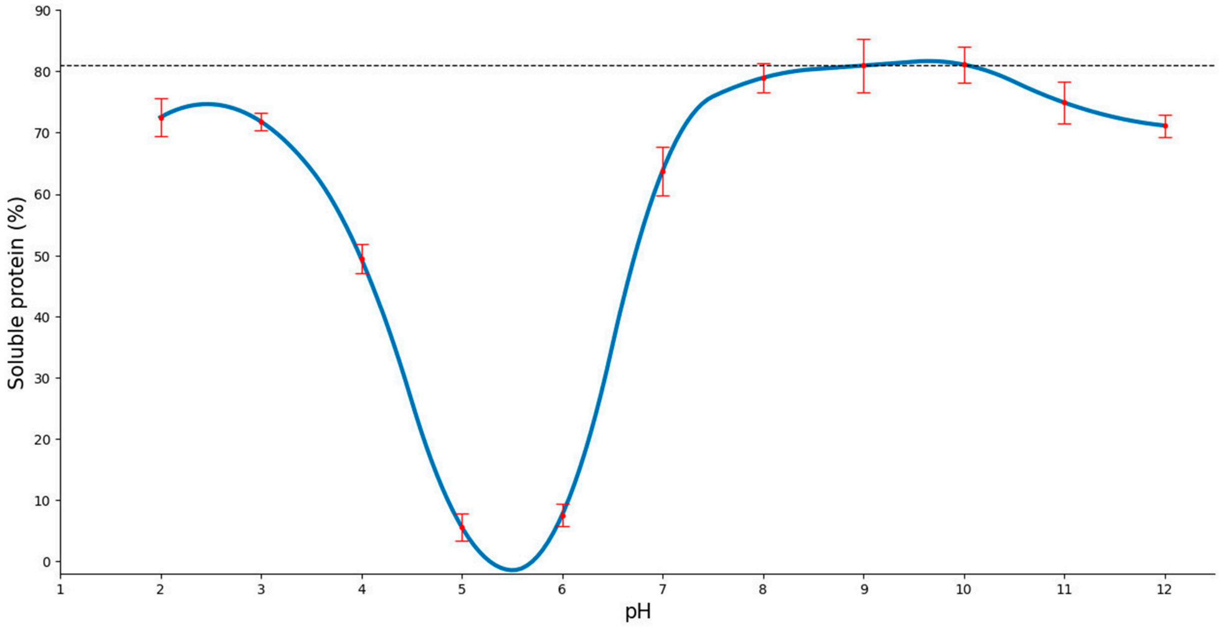

Supplementary figure S1. pH-solubility profiles of protein isolate from cotyledon flour. The percentage of soluble protein was calculated based on the total protein.

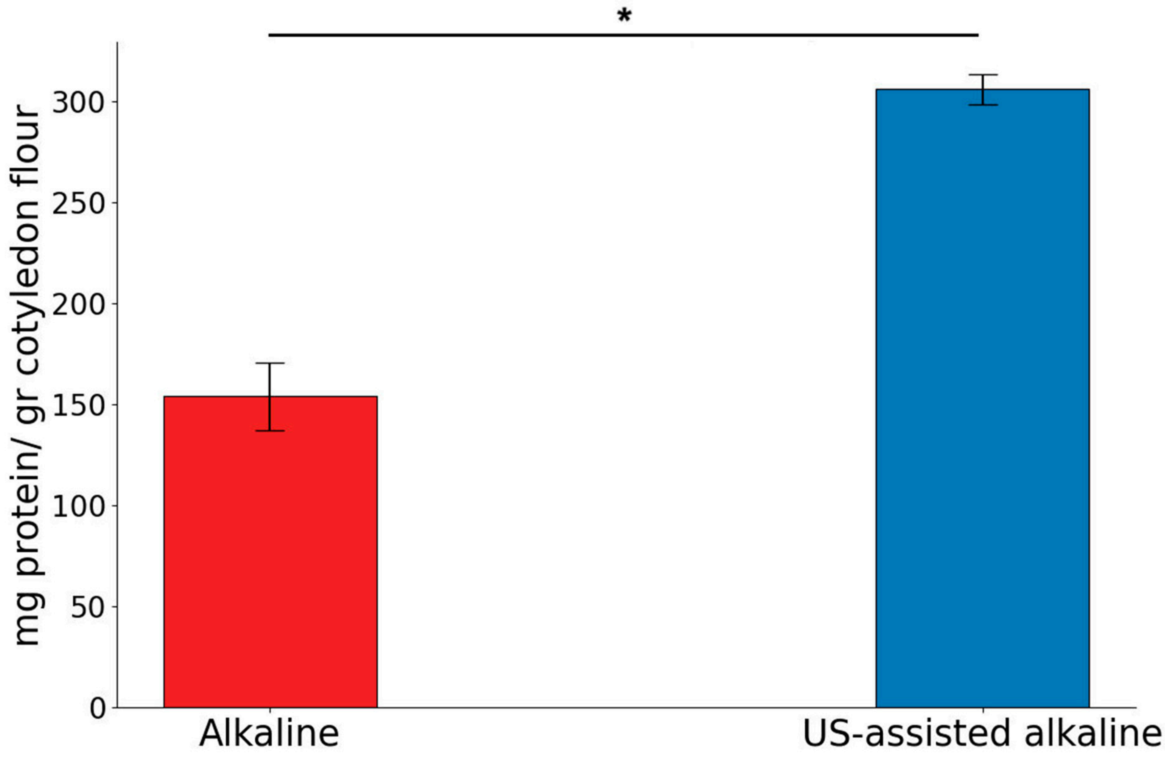

Supplementary figure S2. Comparison of alkaline extraction vs ultrasound-assisted alkaline method. The results are expressed as mg of soluble protein per gram of cotyledon flour.

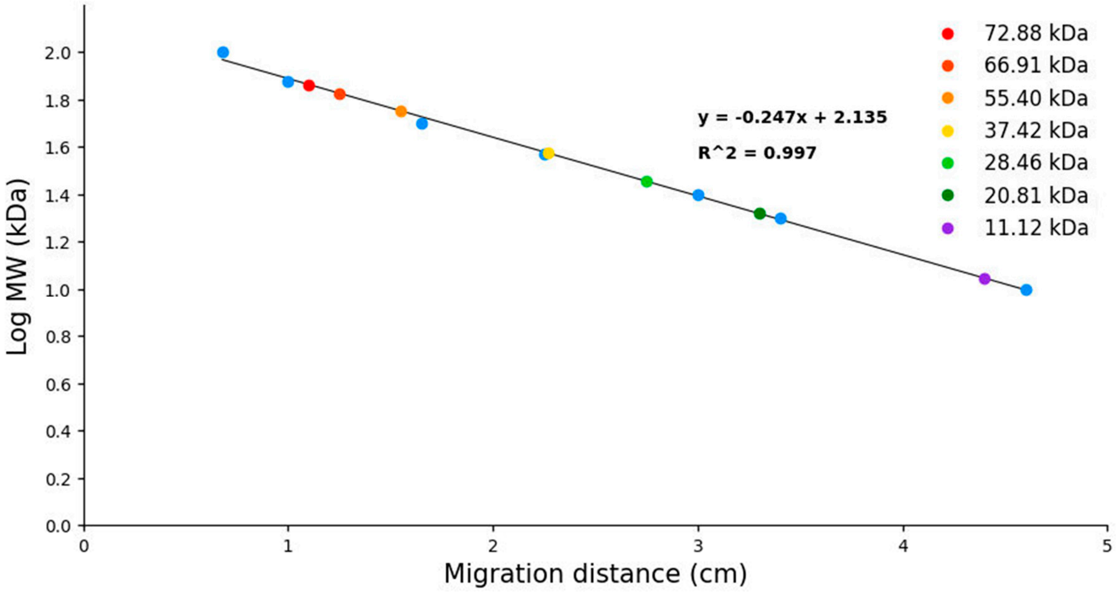

Supplementary figure S3. Plot of the relative mobility (Rm) of the protein with respect to the log of the molecular weight. Blue dots correspond to the molecular weight standard.
